# Transcriptomic Profiling of Primary Microglia: Effects of miR-19a-3p and miR-19b-3p on Microglia Activation

**DOI:** 10.3390/ijms251910601

**Published:** 2024-10-01

**Authors:** Faezeh Sahebdel, Aliabbas Zia, Hector Ramiro Quinta, Leslie R. Morse, Julie K. Olson, Ricardo A. Battaglino

**Affiliations:** 1 Department of Rehabilitation Medicine, School of Medicine, University of Minnesota, Minneapolis, MN 55455, USA; faezeh.sahebdel@gmail.com; 2 Research Center of Centre Hospitalier Universitaire Sainte-Justine, Montreal, QC H3T1C5, Canada; 3 Department of Pharmacology, Université de Montréal, Montreal, QC H3T1J4, Canada; 4 National Scientific and Technical Research Council (CONICET), Ciudad Autonoma de Buenos Aires C1425FQB, Argentina; 5 Laboratorio de Medicina Experimental, “Dr. Jorge Toblli”, Hospital Aleman, Ciudad Autonoma de Buenos Aires C1425FQB, Argentina; 6 Department of Physical Medicine and Rehabilitation, Miller School of Medicine, University of Miami, Miami, FL 33136, USA; 7 Uhealth and Jackson Health Systems, Lynn Rehabilitation Center, Miami, FL 33136, USA; 8 Department of Diagnostics and Biological Sciences, School of Dentistry, University of Minnesota, Minneapolis, MN 55455, USA; 9 Department of Orthopaedics, Miller School of Medicine, University of Miami, Miami, FL 33136, USA

**Keywords:** microRNA-19a-3p, microRNA-19b-3p, microglia, neuroinflammation, spinal cord injury

## Abstract

Neuropathic pain resulting from spinal cord injury (SCI) is a significant secondary health issue affecting around 60% of individuals with SCI. After SCI, activation of microglia, the immune cells within the central nervous system, leads to neuroinflammation by producing pro-inflammatory cytokines and affects neuropathic pain. This interplay between inflammation and pain contributes to the persistent and intense pain experienced by many individuals with SCI. MicroRNAs (miRs) have been critical regulators of neuroinflammation. Previous research in our laboratory has revealed upregulation levels of circulating miR-19a and miR-19b in individuals with SCI with neuropathic pain compared to those without pain. In this study, we treated primary microglial cultures from mice with miR-19a and miR-19b for 24 h and conducted RNA sequencing analysis. Our results showed that miR-19a and miR-19b up- and downregulate different genes according to the volcano plots and the heatmaps. miR-19a and miR-19b regulate inflammation through distinct signaling pathways. The results showed that miR-19a promotes inflammation via toll-like receptor signaling, TNF signaling, and cytokine–cytokine receptor interactions, while miR-19b increases inflammatory responses through the PI3K-Akt signaling pathway, focal adhesion, and extracellular matrix receptor interactions. The protein–protein interaction (PPI) networks used the STRING database to identify transcription factors associated with genes up- or downregulated by miR-19a and miR-19b. Key transcription factors, such as STAT1, STAT2, and KLF4 for miR-19a, and Nr4a1, Nr4a2, and Nr4a3 for miR-19b, were identified and revealed their roles in regulating neuroinflammation. This study demonstrates that miR-19a and miR-19b modulate diverse patterns of gene expression, regulate inflammation, and induce inflammatory responses in microglia.

## 1. Introduction

Spinal cord injury (SCI) is a challenging disorder with no current effective treatment. Neuroinflammation is the primary pathogenesis of secondary damage following SCI that results in tissue loss and neurological damage [1,2]. The most critical stage in pathophysiology SCI is known as the secondary injury. This is the damaging process that happens by abnormal molecular signaling, inflammation, changes in blood vessels, and cellular damage [3]. Neuroinflammation is a localized form of inflammation that can affect both the peripheral nervous system (PNS) and central nervous system (CNS) [4]. Neuroinflammation is characterized by increased vascular permeability, leukocyte infiltration, glial cell activation, and increased production of inflammatory mediators (cytokines and chemokines) [4]. Neuroinflammation activates both peripheral and central glia such as Schwann cells, satellite glial cells (SGCs), microglia, astrocytes, and oligodendrocytes [5,6].

Microglia are the resident macrophages of the CNS and act as the brain’s first line of defense by phagocytosing harmful pathogens and cellular debris [7]. In the quiescent state, microglia are dynamic surveyors of the CNS environment and capable of activating pro-inflammatory responses such as cytokine signaling, debris phagocytosis, antigen presentation, and immune cell recruitment. Microglia respond quickly to spinal cord damage by extending their processes to the lesion site [8,9]. Chronically activated microglia in neurodegenerative processes can produce inflammatory cytokines, including tumor necrosis factor-α (TNF-α), interleukin-6 (IL-6), and interleukin-1β (IL-1β), as well as reactive oxygen species (ROS) and excitotoxins like glutamate. Microglia produce chemicals that have a beneficial and detrimental effect on the microenvironment [7].

MicroRNAs (miRs) are small, non-coding RNAs (18–25 nucleotides) that negatively regulate gene expression after transcription [10]. miRs bind to complementary regulatory RNA sequences and cause mRNA decay or translational repression, which finally affects gene expression. Humans have more than 5000 miRs, and each miR can bind up to 200 RNAs. Important pro-inflammatory (microRNA-155, microRNA-27b, and microRNA-326), anti-inflammatory (microRNA-124, microRNA-146a, microRNA-21, and microRNA-223), and mixed immunomodulatory (let-7 family) miRs control neuroinflammation in different diseases such as Alzheimer’s, multiple sclerosis, spinal cord injury, and ischemic stroke [11]. A study showed that some mRNAs of inflammatory mediators, including intercellular adhesion molecule 1 (ICAM-1), IL-1β, and TNF-α, were possible targets of microRNA-181a, microRNA-411, microRNA-99a, microRNA-34a, microRNA-30c, microRNA-384-5p, microRNA-30b-5p [12], and microRNA-486 [13], which were downregulated after SCI. Other studies showed that microRNA-17, microRNA-20 [14], and microRNA-124a were all upregulated after SCI, while microRNA-124a was downregulated at 1 and 7 days after SCI [15].

MicroRNAs (miRs) play a critical role in regulating microglia functions at both molecular and mechanistic levels. They control gene expression post-transcriptionally by binding to complementary sequences in the 3′ untranslated region (3′ UTR) of target messenger RNA (mRNAs), leading to either mRNA degradation or translational repression. When miRs bind, they either repress translation or promote mRNA degradation, depending on the degree of complementarity. If there is a complete complementarity, the mRNA is degraded by the RNA-induced silencing complex (RISC), while in cases of partial complementarity, translation is inhibited without degrading the mRNA. Through these mechanisms, miRs regulate gene expression and contribute to various cellular processes such as differentiation, proliferation, and apoptosis [16]. For example, miR-124 maintains microglia quiescence by targeting CCAAT/enhancer-binding protein alpha (C/EBP-α) and preventing pro-inflammatory gene expression, while miR-155 promotes microglia activation by downregulating suppressor of cytokine signaling 1 (SOCS1)**.** SOCS1 is a negative regulator of cytokine signaling, which enhances nuclear factor kappa-light-chain-enhancer of activated B cells (NF-κB)-driven inflammation. Additionally, miR-146a acts as a feedback regulator, suppressing Interleukin-1 receptor-associated kinase 1 (IRAK1) and TNF receptor-associated factor 6 (TRAF6), reducing excessive microglia activation and inflammation. These miRs fine-tune the balance between microglial activation and resolution of inflammation, contributing to neuroinflammation and neurodegeneration in various CNS conditions [17,18,19].

It has been shown that miRs play crucial roles in microglia activation, inflammation, and differentiation/polarization. miR-based therapeutics may be a possible approach to enhance microglia function and control genes/pathways in neuropathogenesis by regulating gene expression [20]. A group of RNAs can be regulated by certain immunomodulatory miRs that raise the possibility of effective miR-based therapeutic methods for neuroinflammatory diseases. For example, pro-inflammatory responses may be induced by miRs that can limit the translation of several cellular anti-inflammatory proteins. Numerous studies have shown the precise functions of particular miRs in microglia [21]. Microglia are significantly active in most CNS pathological states, and the change from a resting to an activated state may be crucial for neuropathogenesis [22].

In a recent study conducted in our laboratory [23], the authors found 71 miRs that are differently expressed in people living with chronic SCI and neuropathic pain versus in people who do not experience pain. Two related miRs, hsa-miR-19a-3p and hsa-miR-19b-3p, showed strong differences between pain and non-pain groups and were significantly raised in people with neuropathic pain linked to SCI [23]. These results demonstrate the potential of miRs as therapeutic targets for neuropathic pain and neuroinflammatory disorders. The identification of miRs that are associated with neuropathic pain and play a role in regulating inflammatory processes leads to the development of miR-based therapeutics that are beneficial for persons living with spinal cord injury.

The aim of this study is to investigate the potential role of miR-19a and miR-19b in microglia activation and neuroinflammation by analyzing transcriptomic profiling of primary microglia. We hypothesized that miR-19a and miR-19b promote microglia activation by affecting the expression of genes involved in activation. Our research suggests the hypothesis that miR-19a and miR-19b change gene expression patterns and trigger diverse inflammatory responses in microglia. This study aims to uncover the role miRs play in neuroinflammation and microglia activation.

## 2. Results

### 2.1. MiR-19a and miR-19b up- and Downregulated the Expression of Different Genes According to Volcano Plot and Heatmaps

**Figure 1:**
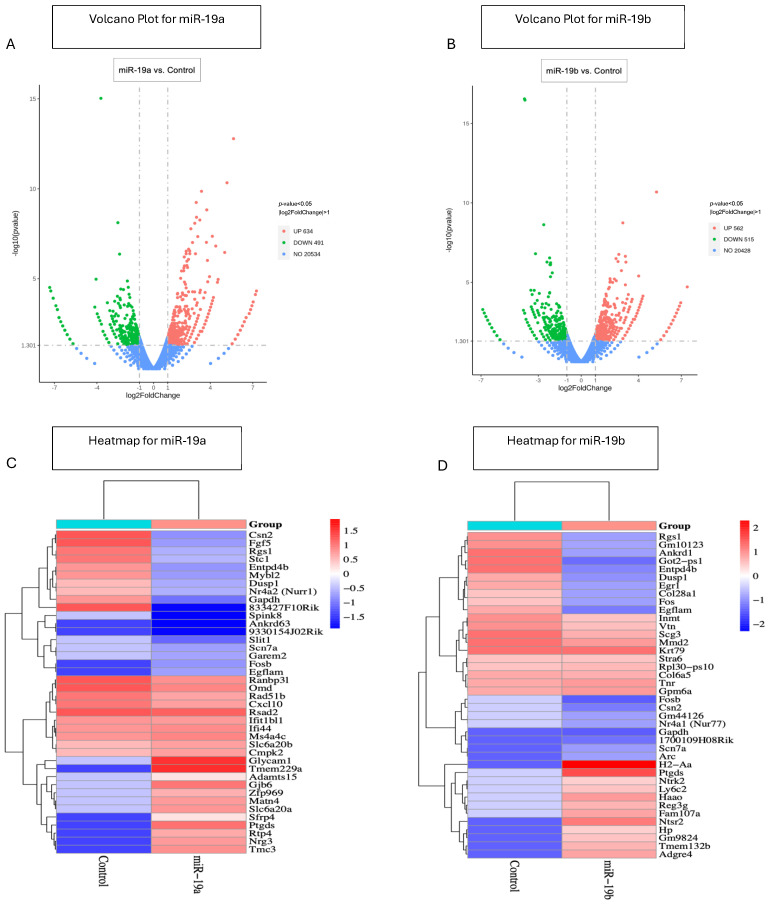
**Volcano plot for Differentially expressed genes (DEGs) and Heatmaps for the top 20 DEGs of genes up- or downregulated by miR-19a and miR-19b.** Volcano plot showed 21,659 DEGs, 634 upregulated, and 491 downregulated genes in microglia transfected with miR-19a compared to the control group (**A**). DEGs were also analyzed between microglia transfected with miR-19b and controls, and 21,505 total DEGs, 562 upregulated, and 515 downregulated genes could be found (**B**). The red dots represent upregulation, the green dots show downregulation, and the blue dots show no significant changes. Heatmaps show the upregulation and downregulation of genes in microglia transfected with miR-19a (**C**) and miR-19b (**D**) compared with the control. In heatmaps, red indicates upregulation and blue indicates downregulation of genes.

Primary microglia were isolated from the brains of 2-day-old newborn SJL/J mice. Mixed glial cultures were maintained for 14 days, followed by separation of the microglia using an orbital shaker. Microglia were then stimulated with miR-19a and miR-19b mimics for 24 h to assess their role in regulating neuroinflammatory processes. Transfecting microglia cells with miR-19a or miR-19b causes considerable alterations in gene expression, resulting in different gene expression versus control, non-transfected cells. Differentially expressed genes (DEGs) were compared between microglia transfected with miR-19a and control. In all, 21,659 DEGs were detected according to the volcano plot. Microglia transfected with miR-19a showed 634 upregulated and 491 downregulated genes compared to the control group (Figure 1A). DEGs were also compared between microglia transfected with miR-19b and control, and 21,505 DEGs were detected. Microglia transfected with miR-19b showed 562 significantly upregulated and 515 significantly downregulated genes compared to control group (Figure 1B). Among all these genes, we chose the 20 most upregulated and downregulated ones expressed in microglia transfected with miR-19a or miR-19b compared to the control. Figure 1C,D display heatmaps showing the names of the genes that are upregulated and downregulated by miR-19a and miR-19b, respectively, compared to the control group. These genes were selected based on their log2 fold changes. Specifically, we chose the 20 genes with the highest and 20 with the lowest fold changes in microglia transfected with both miR-19a (Figure 1C) and miR-19b (Figure 1D).

### 2.2. MiR-19a Increased the Inflammatory Genes and Signaling Pathways in Microglia

**Figure 2:**
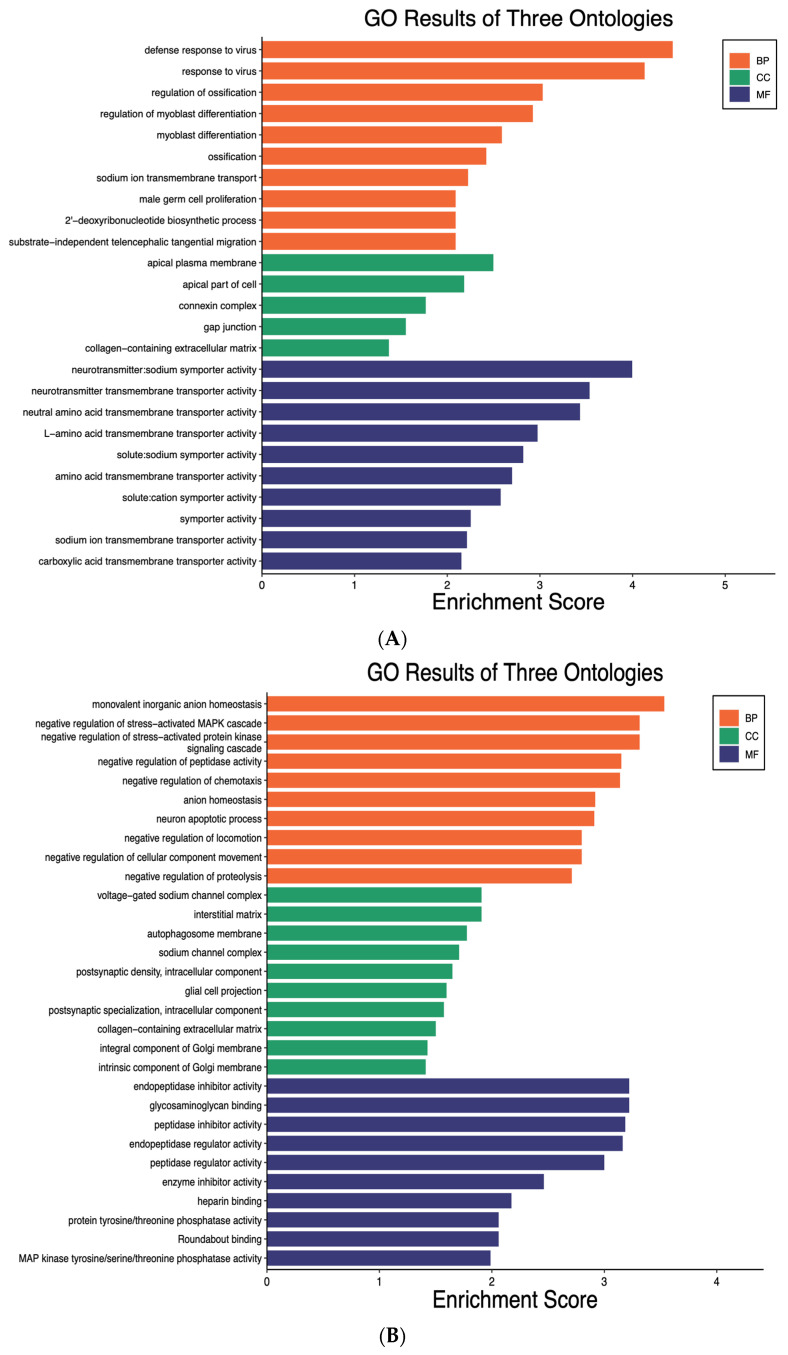

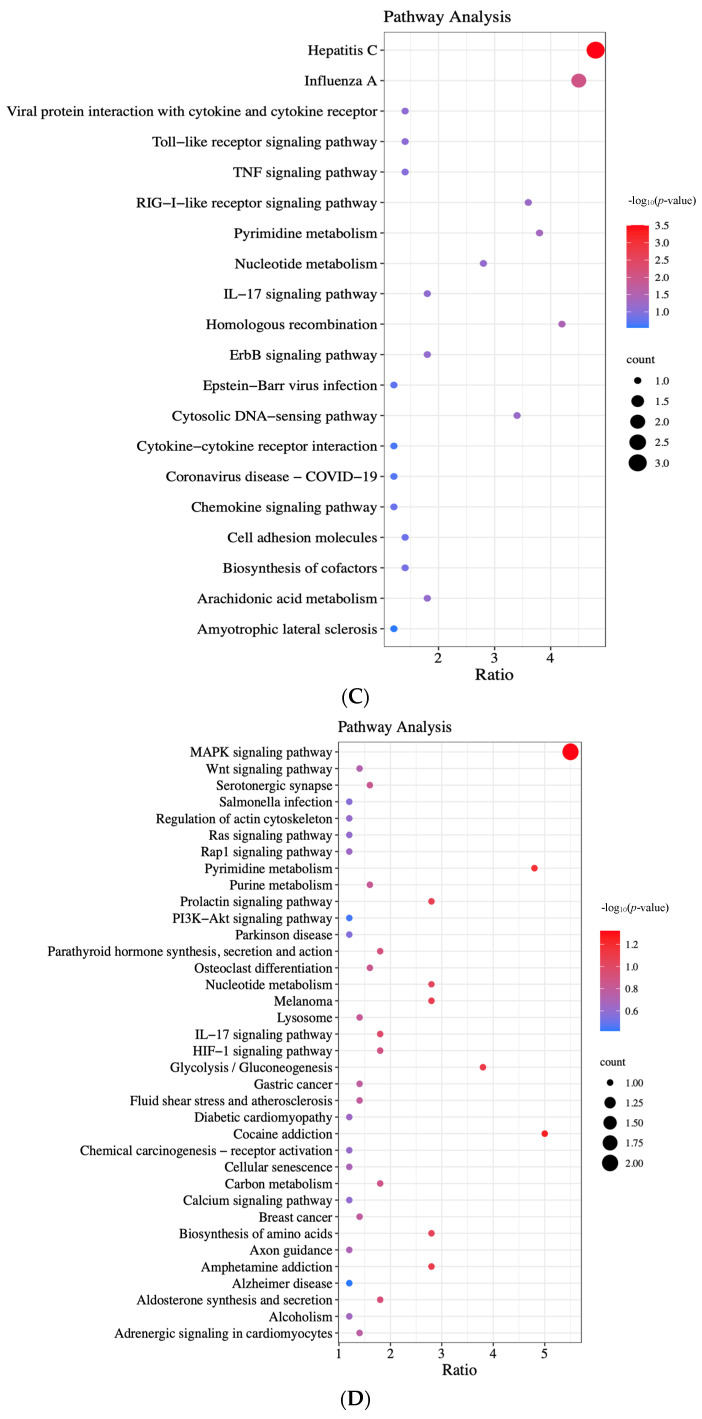
**Gene Ontology (GO) enrichment analysis and Kyoto Encyclopedia of Genes and Genomes (KEGG) Pathways for the 20 most upregulated and downregulated DEGs by miR-19a in microglia.** The top ten highly enriched GO keywords in biological process (BP), cellular component (CC), and molecular function (MF) branches (*p* < 0.05) were shown (**A**,**B**). DEGs are differentially expressed genes, whereas GO stands for gene ontology. The pathway analysis with KEGG shows the important pathways with a *p*-value less than 0.05, and the *y*-axis represents the pathway name, while the *x*-axis represents the ratio of each pathway (**C**,**D**). The bubble size represents the number of genes. The color bar reflects the −log10^(*p*-value)^, with red representing greater value and blue representing lower value.

We first determined the number of DEGs (adjusted *p* < 0.05, |log2 fold change| > 1) in the microglia transfected with miR-19a and miR-19b compared to the control group. We focused on the top 20 upregulated and downregulated genes in the transfected groups versus control. To annotate these genes in different biological pathways, we performed gene ontology (GO) and Kyoto Encyclopedia of Genes and Genomes (KEGG) analysis. Figure 2A,B, respectively, display the results of GO enrichment analysis of DEG for the 20 most upregulated and downregulated genes between transfected microglia with miR-19a and control. 

Through GO analysis of the 20 most upregulated genes by miR-19a in the annotations of biological progress (BP), the top three most significant processes were defense response to virus, response to virus, and regulation of ossification. One study showed that the virus caused an imbalance of type I IFN responses and inflammation in COVID-19, and it shows the relation between virus and inflammation [24]. In terms of the cellular components (CCs), the most significant annotations were apical plasma membrane, apical part of cell, and connexin complex. As for analysis of molecular function (MF), the top three most significant functions were neurotransmitter sodium symporter activity, neurotransmitter transmembrane transporter activity, and neutral amino acid transmembrane transporter activity (Figure 2A). Through GO analysis of the 20 most downregulated genes by miR-19a in the annotations of biological progress (BP), the top three most significant processes were monovalent inorganic anion homeostasis, negative regulation of stress-activated MAPK cascade, and negative regulation of stress-activated protein kinase signaling cascade. In terms of the cellular components, the most significant annotations were voltage-gated sodium channel complex, interstitial matrix, and autophagosome membrane. As for analysis of MF, the top three most significant functions were endopeptidase inhibitor activity, glycosaminoglycan binding, and peptidase inhibitor activity (Figure 2B). 

### 2.3. MiR-19b Increased the Inflammatory Genes and Signaling Pathways in Microglia

**Figure 3:**
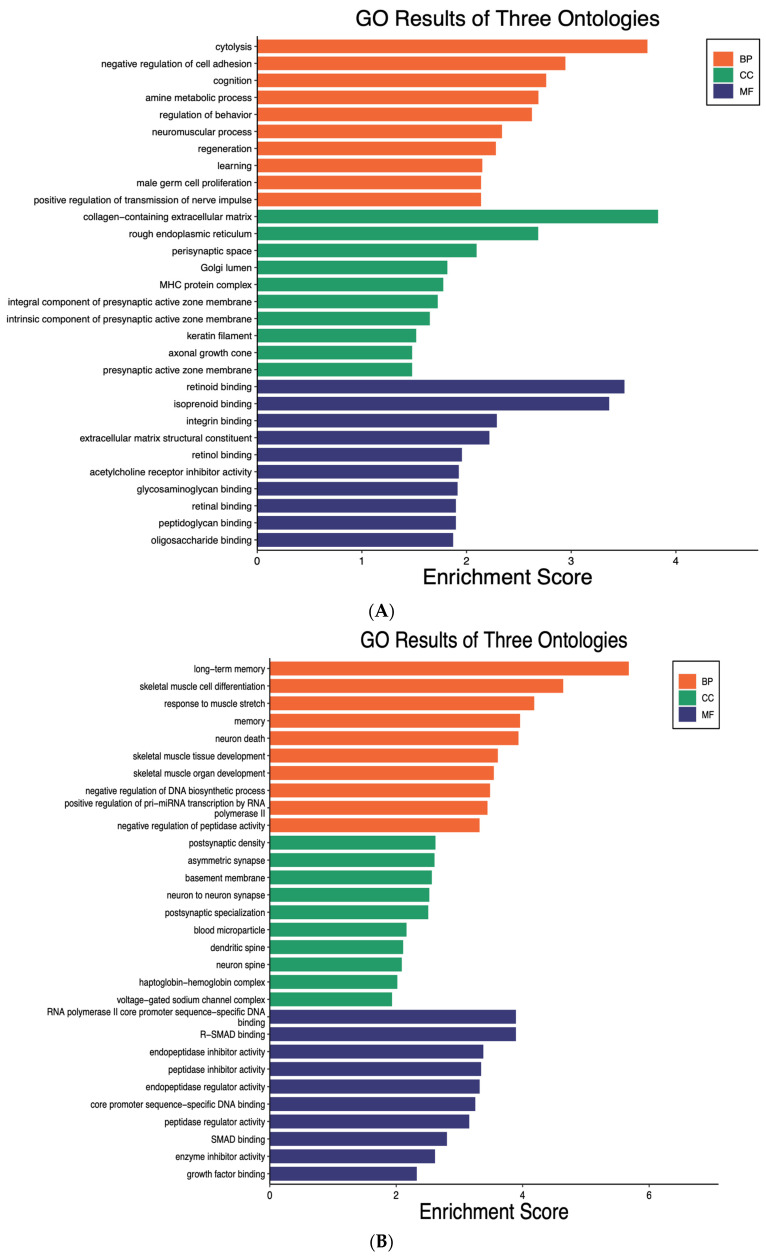

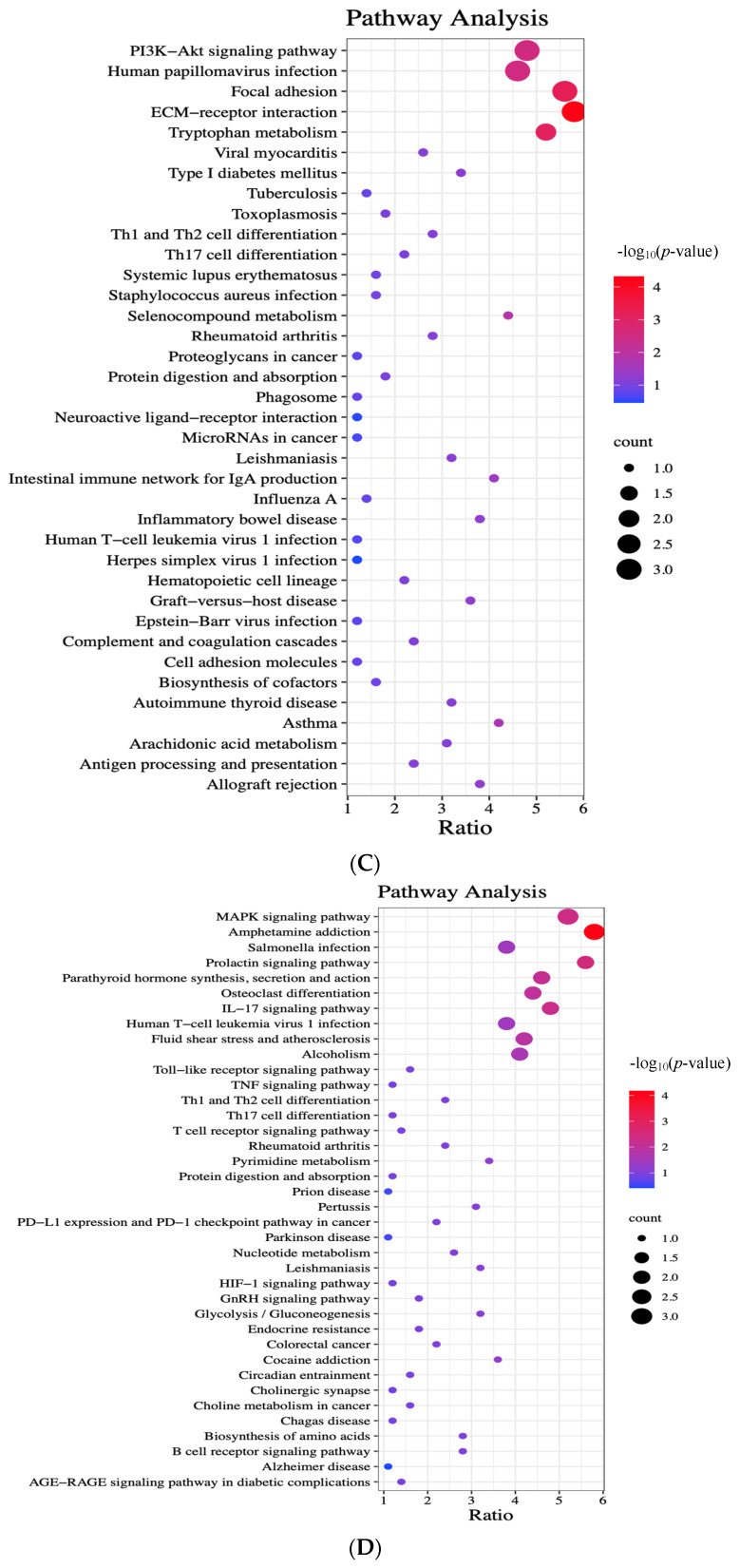
**Gene Ontology (GO) enrichment analysis and Kyoto Encyclopedia of Genes and Genomes (KEGG) pathways for the 20 most upregulated and downregulated DEGs by miR-19b in microglia.** The top ten highly enriched GO keywords in biological process (BP), cellular component (CC), and molecular function (MF) branches (*p* < 0.05) were shown (**A**,**B**). DEGs are differentially expressed genes, whereas GO stands for gene ontology. The pathway analysis with KEGG shows the important pathways with a *p*-value less than 0.05, and the *y*-axis represents the pathway name, while the *x*-axis represents the ratio of each pathway (**C**,**D**). The bubble size represents the number of genes. The color bar reflects the −log10^(*p*-value)^, with red representing greater value and blue representing lower value.

Through GO analysis of the 20 most upregulated genes by miR-19b in the annotations of BP, the top three most significant processes were cytolysis, negative regulation of cell adhesion, and cognition. In terms of cellular components, the most significant annotations were collagen-containing extracellular matrix, rough endoplasmic reticulum, and perisynaptic space. As for analysis of MF, the top three most significant functions were retinoid binding, isoprenoid binding, and integrin binding (Figure 3A). Through GO analysis of the 20 most downregulated genes by miR-19b in the annotations of BP, the top three most significant processes were long-term memory, skeletal muscle cell differentiation, and response to muscle stretch. In terms of cellular components, the most significant annotations were post-synaptic density, asymmetric synapse, and basement membrane. As for analysis of MF, the top three most significant functions were RNA polymerase II core promotor sequence-specific DNA binding, R-SMAD binding, and endopeptidase inhibitor activity (Figure 3B). 

KEGG pathway analysis indicated that the 20 most upregulated DEGs by miR-19b were involved in important neuroinflammatory signaling pathways, including focal adhesion, ECM-receptor interaction, microRNAs in cancer, human T-cell leukemia virus 1 infection, and inflammatory bowel disease (Figure 3C). One study found that ECM plays a key role in neuroinflammation following acute injury and chronic inflammatory disorders of the central nervous system [25]. KEGG pathway analysis indicated that the 20 most downregulated DEGs by miR-19b were mainly involved in important anti-inflammatory signaling pathways such as MAPK signaling pathway and IL-17 signaling pathway (Figure 3D). It shows that genes downregulated by miR-19b were involved in the regulation of anti-inflammatory and neuroprotection effects on the cells.

### 2.4. Protein–Protein Interaction (PPI) Network among Major TFs Related to miR-19a and miR-19b up- and Downregulated Genes

**Figure 4:**
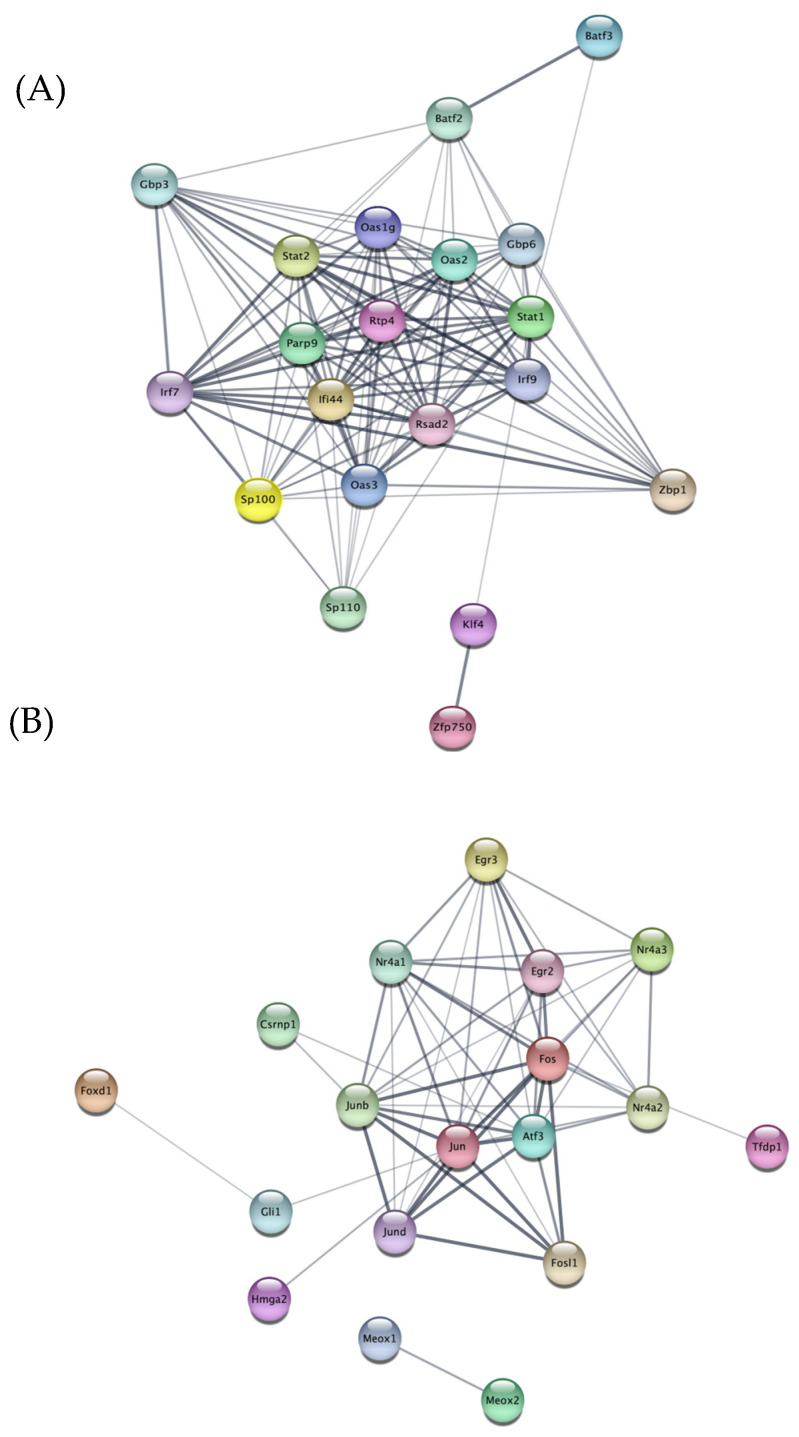
**Protein–protein interaction (PPI) network analysis of main transcription factors involved in the 20 most upregulated and downregulated genes by miR-19a.** STRING Protein–Protein Interaction Network (Version 12.0) tool creates networks of interactions between key TFs regarding to genes that are upregulated (**A**) or downregulated (**B**) by miR-19a. The line thickness indicates the strength of the interaction.

**Figure 5:**
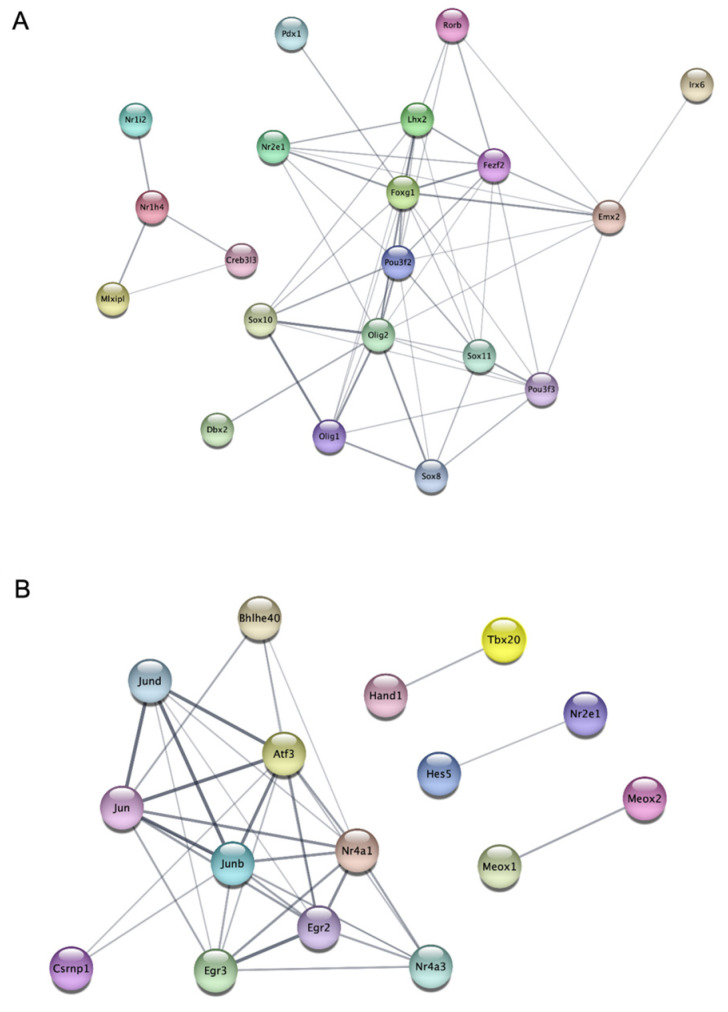
Protein–protein interaction (PPI) network analysis of main transcription factors involved in the 20 most upregulated and downregulated genes by miR-19b. STRING Protein–Protein Interaction Network (Version 12.0) tool creates networks of interactions between key TFs regarding to genes that are upregulated (**A**) or downregulated (**B**) by miR-19b. The line thickness indicates the strength of the interaction.

We used the STRING database and created a protein–protein interaction (PPI) network that involves important transcription factors that are related to genes that are either up- or downregulated by miR-19a or miR-19b. An analysis of protein–protein interaction networks of genes up- or downregulated by miR-19a (Figure 4) or miR-19b (Figure 5) revealed some transcription factors as listed by their rankings. The tables show specific transcription factors, by their ranks up to 25th, up- or downregulated by miR-19a (Table 1) and miR-19b (Table 2) according to the protein–protein interaction networks. 

An analysis of protein–protein interaction networks revealed that miR-19a upregulates some protein levels related to inflammation (Figure 4A). Some of these proteins such as STAT2, STAT1, and KLF4 regulate neuroinflammation [26,27].

Protein–protein interaction (PPI) network between major TFs related to miR-19a downregulated genes showed some proteins, such as Nr4a1, Nr4a2, and Nr4a3, are important in regulating inflammation (Figure 4B). Other studies showed that these proteins have important roles in inflammation [28,29].

There are key transcription factors associated with genes that are upregulated by miR-19a, such as Nr2e1 and Olig2, and play essential roles in inflammation (Figure 5A). Other studies found that they have an effect on inflammation [30,31,32,33,34]. There are some important transcription factors related to genes that are downregulated by miR-19b, such as Nr4a1, Nr4a2, Nr4a3, and JunB (Figure 5B). Other studies found that these TFs can regulate inflammation [33,34].

## 3. Discussion

The combined effects of microRNA on microglia activity are not fully understood. To address this gap in the knowledge, we transfected primary microglia with miR-19a and miR-19b and performed a transcriptomic profile analysis. The results of this study show that miR-19a and miR-19b affect the expression of genes that regulate neuroinflammation.

The heat maps show a significant difference in the 20 most upregulated and downregulated genes in microglia transfected with miR-19a (Figure 1C) or miR-19b (Figure 1D) compared to the control group. Genes seen as upregulated are mostly involved in inflammation, while downregulated genes mainly regulate anti-inflammation. For example, our results showed that Nr4a2 (Nurr1) was downregulated by miR-19a. Downregulation of Nurr1 due to miR-19a shows that microglia play a critical role in anti-inflammation and neuroprotection. One study showed that Nurr1 inhibited the expression of pro-inflammatory cytokine genes [35]. Another gene that was downregulated in response to miR-19b was Nr4a1 (Nur77). One study suggested that overexpression of Nur77 or pharmacological activation of Nur77 decreased LPS-induced inflammatory signaling in microglia, but the loss of Nur77 functions increased LPS-induced pro-inflammatory gene expression and the resulting neurotoxicity [36]. Another gene that was shown to be upregulated by miR-19a was CXCL10. One study suggested that CXCL10 actively contributes to the initial phase of microglia activation [37], confirming our data. 

Our results showed that the KEGG pathways of the 20 most upregulated DEGs by miR-19a were found to be central to important neuroinflammatory signaling pathways including viral protein interaction with cytokine and cytokine receptor, Toll-like receptor signaling pathway, TNF signaling pathway, cytokine–cytokine receptor interaction, and chemokine signaling pathway (Figure 2C). The 20 most downregulated DEGs by miR-19a, on the other hand, were related to important anti-inflammatory signaling pathways, including MAPK signaling pathway, pyrimidine metabolism, PI3K-Akt signaling pathway, (Figure 2D). These observations show that genes downregulated by miR-19a were involved in the regulation of anti-inflammation and neuroprotection effects on the cells. One study showed that Hesperetin reduces neuroinflammation in microglia by inhibiting inflammatory cytokines and MAPK pathways [38]. TREM2 activation can reduce neuroinflammation through the PI3K/Akt signaling pathway, which improves postoperative cognitive impairment in mice [39]. One study revealed that in the absence of pathogens, TLR2 and TLR4 play crucial roles in coordinating post-injury sequelae and, possibly, controlling inflammation and gliosis following SCI [40]. Another study showed that the activation of TLRs plays a vital role in producing neuroinflammatory immune responses [41]. Moreover, we have revealed for the first time that miR-19a upregulated cytokines such as TNF-α. One study showed that TNF-α triggers microglia activation via the NF-κB signaling pathway and that miR-342 is a crucial mediator in activating it [42]. Our results also showed upregulation of chemokine signaling such as C-C motif chemokine ligand 2 (CCL2), C-X-C motif chemokine ligand 8 (CXCL8), and C-X-C motif chemokine ligand 10 (CXCL10) in response to miR-19a. Several papers showed that chemokine signaling regulates neuroinflammation in the CNS [43,44] and it confirmed our data.

Our results revealed the KEGG pathways of the 20 most downregulated DEGs by miR-19a were found to be central to important anti-inflammatory signaling pathways. One of these pathways was the MAPK signaling pathway. One study showed that dexmedetomidine reduced neuroinflammation by changing microglial M1/M2 polarization via the MAPK/ERK pathway [45]. Another pathway was the Ras signaling pathway. One study showed that Nurr1 modulates RasGRP1 expression at the transcriptional level and functions as an anti-inflammatory mediator in neuroinflammation [35].

It is of much interest that our results showed that the KEGG pathways of the 20 most upregulated DEGs by miR-19b were found to be also central to important inflammatory pathways. One of these pathways was ECM-receptor interaction that was upregulated in response to miR-19b. One study showed that ECM has important functions in neuroinflammation after acute injury and chronic inflammatory diseases of the central nervous systems [25]. Our results showed that the PI3k/Akt signaling pathway will be upregulated in response to miR-19b. The results of one study suggested that TLR4 activates the PTEN/PI3K/AKT/NF-kB signaling pathway in rat hippocampus neurons, leading to a neuroinflammatory response [46], and will confirm our data.

The KEGG pathway of the 20 most downregulated DEGs by miR-19b showed being effective in anti-inflammation. For example, our results showed that the MAPK signaling pathway downregulated in response to miR-19b. Another study revealed that dexmedetomidine inhibits neuroinflammation by changing microglial M1/M2 polarization via the MAPK/ERK pathway [45].

Using the STRING database, we developed a protein–protein interaction network containing major transcription factors that were activated by genes either up- or downregulated by miR-19a or miR-19b. Protein–protein interaction of the 20 most up-and downregulated genes by miR-19a and miR-19b in microglia showed a list of proteins that will be activated. We showed that the transcription factors that were shown in PPI analysis are important in regulating inflammation. 

For example, one of the proteins with the first highest rank of interaction with other proteins that was shown in the PPI network for genes upregulated by miR-19a was signal transducer and activator of transcription 2 (STAT2). One study showed that STAT2 has a role in inflammation by triggering autocrine responses to IFN-α/β and inducing inflammatory chemokines and cytokines. Therefore, our results could confirm the previous studies showing the effect of STAT2 in regulating inflammation [47].

Another protein upregulated by miR-19a in this list was STAT1. A recent study has shown that STAT1 participates in TLR signal transduction and inflammatory reactions [26]. Another protein in this list was KLF4. One study showed that KLF4 has diverse roles in carcinogenesis; overexpression of Klf4 in esophageal epithelial cells promotes inflammation [27]. Our findings also revealed that KLF4, which is linked to inflammation in the previous research, was one of the proteins identified in PPI for miR-19a-upregulated genes. 

There is another list of transcription factors relating to the genes that were downregulated by miR-19a that are involved in modulating inflammation. Different studies have shown that Nr4a1 has an anti-inflammatory effect on the cells. For instance, the loss of Nr4a1 (Nur77) activates macrophages to have an inflammatory phenotype and increases the risk of atherosclerosis [28]. Another study showed that Nr4a2 is expressed in microglia and astrocytes and inhibits the production of pro-inflammatory mediators. It protects DA neurons from inflammation-mediated death [29]. Furthermore, Nr4a2 regulates many signaling pathways to protect neurons [29]. Another study revealed that Nr4a3 may reduce myocardial inflammatory responses through JAK2-STAT3/NF-kB signaling and make it a potential therapeutic target for heart remodeling following myocardial infarction [33]. Some transcription factors associated with miR-19b-upregulated genes, such as Nr2e1 and Olig2, play key roles in inflammation. It was interesting to know that Nr2e1 had the first rank in the PPI analysis for miR-19b-upregulated genes. One study showed that increased Nr2e1 levels may be linked to inflammation and lipid and glucose metabolism disorders in diabetic individuals [30]. Another study showed that Nr2e1 could function as a target for improving insulin sensitivity and inflammation in obesity and associated problems [31]. Olig2 is important in regulating inflammation and is shown as one of the proteins on the list of miR-19b upregulation genes. One study showed that Olig2+ glial precursor cells play an important function in the adult CNS by connecting autoimmune inflammation and glial scar formation [32], and our data confirms it. 

Some key transcription factors like NF-κB and activator protein 1 (AP-1) are associated with genes downregulated by miR-19b, such as Nr4a1, Nr4a2, Nr4a3, and JunB. After a myocardial infarction [48], Nr4a3 reduces the size of the infarct, enhances left ventricular function, and lessens the inflammatory response that was triggered. Furthermore, through JAK2-STAT3/NFkB signaling, Nr4a3 inhibits the post-AMI inflammatory response [33]. Another study showed that JunB inhibits alternative CD4+ T-cell programs and increases Th17 cell identity in the process of inflammation [34]. In conclusion, our data support the concept that the two miRs cause distinct patterns of gene expression and promote different inflammatory profiles in microglia. This study shows the significance of these miRs in microglia activation and neuroinflammation by looking into their effects on changes in the transcription factor expression in microglia.

## 4. Materials and Methods

### 4.1. Microglia Cultures

Primary microglia cells were isolated from SJL/J mice as previously described [49]. In brief, newborn mouse brains were dissected, minced, and digested to yield a cell suspension. Cells were grown as a mixed glia culture for 14 days. The cultures were then shaken on an orbital shaker for 24 h to separate the microglia. Microglia were grown in poly-D-lysine-coated 24 well plates with DMEM high-glucose media (Gibco, Waltham, MA, USA), 20% FBS, and 3 ng/mL rGM-CSF (R&D Systems, Minneapolis, MN, USA). 

### 4.2. Microglia Activation, Transfection, and RNA Isolation

Microglia cells were transfected and stimulated with miR-19a mimic and miR-19b mimic (Qiagen, Hilden, Germany) for 24 h at 37 °C. Control cultures were left untransfected. miRs were transfected using Lipofectamine RNAiMAX (Invitrogen, Waltham, MA, USA, #13778-030) according to the manufacturer’s protocol (Qiagen). After incubating the microglia cells for 24 h, the SV Total RNA Isolation kit (Promega, Madison, WI, USA, #Z3100) was used to lyse and isolate total RNA according to the manufacturer’s instructions.

### 4.3. RNA-Seq Library Construction and Sequencing 

The total RNA of the microglia was extracted. Three biological replicates were carried out for each condition. Library preparation and mRNA sequencing were performed at Novogene using their Illumina Novoseq 6000 platform, and 150 bp paired-end reads were generated.

### 4.4. Quality Control 

Raw data (raw reads) of Fastq files were initially processed via in-house Perl scripts. During this process, the data underwent quality filtering to obtain clean data (clean reads) by removing reads containing adapters, poly-N, and low-quality reads from raw data. Additionally, metrics such as Q20, Q30, and GC content were calculated for the resulting clean reads. Subsequent analyses were performed exclusively on the clean data, ensuring high data quality throughout the downstream analyses.

### 4.5. Reads Mapping to the Reference Genome 

The reference genome and gene model annotation files were directly retrieved from the genome website. At the next step, we used Hisat2 v2.0.5 to build an index of the reference genome, and paired-end clean reads were aligned to it. We selected Hisat2 as the mapping too, since it has the capability to generate a splice junction database from the provided gene model annotation file. According to this feature of Hisat2, we can obtain more accurate mapping results compared to other non-splice mapping tools.

### 4.6. Quantification of Gene Expression Level 

We used Feature Counts v1.5.0-p3 to count the number of reads mapped to individual genes. Subsequently, the fragments per kilobase of transcript sequence per million base pairs sequenced (FPKM) for each gene was computed. FPKM, which represents the expected number of fragments per kilobase of transcript sequence per million base pairs sequenced, accounts for both sequencing depth and gene length in the read count calculation. This method stands as the prevailing approach for estimating gene expression levels due to its comprehensive consideration of these factors.

### 4.7. Differential Expression Analysis 

Differential expression analysis between two conditions/groups was conducted utilizing the DESeq2 R package (version 1.20.0). DESeq2 offers robust statistical algorithms to identify differential expressions in digital gene expression data, employing a model grounded in the negative binomial distribution. Subsequently, the resulting *p*-values underwent adjustment employing the Benjamini and Hochberg method to control the false discovery rate. Genes exhibiting an adjusted *p*-value of ≤0.05 as determined by DESeq2 were designated as differentially expressed. Before conducting the analysis on differential gene expression, the read counts for each sequenced library underwent adjustment by the edgeR program package, employing a single scaling normalization factor. Subsequently, differential expression analysis between two conditions was carried out using the edgeR R package (version 3.22.5). The resulting *p*-values underwent correction using the Benjamini and Hochberg method. Significance thresholds for differential expression were set at a corrected *p*-value of 0.05 and an absolute fold change of 2.

### 4.8. Gene Ontology (GO) and Kyoto Encyclopedia of Genes and Genomes (KEGG) Enrichment Analyses of DEGs

The Gene Ontology (GO) enrichment analysis of differentially expressed genes was conducted using the clusterProfiler R package, which includes correction for gene length bias. GO terms with a corrected *p*-value below 0.05 were deemed significantly enriched by differentially expressed genes. The Kyoto Encyclopedia of Genes and Genomes (KEGG) serves as a database resource for comprehending the high-level functions and utilities of biological systems, spanning from the cellular level to the organism and ecosystem levels. We employed the clusterProfiler R package to assess the statistical enrichment of differentially expressed genes within KEGG pathways. The Reactome database combines different reactions and biological pathways of human model species. Reactome pathways with a corrected *p*-value below 0.05 were identified as significantly enriched by differentially expressed genes. The Disease Ontology [21] database defines the functions of human genes and diseases. DO pathways with a corrected *p*-value below 0.05 were recognized as significantly enriched by differentially expressed genes. Additionally, the DisGeNET database integrates genes associated with human diseases. DisGeNET pathways with a corrected *p*-value below 0.05 were considered significantly enriched by differentially expressed genes. We utilized the clusterProfiler software (version number 4.10.1) to evaluate the statistical enrichment of differentially expressed genes across Reactome, DO, and DisGeNET pathways.

### 4.9. Gene Set Enrichment Analysis 

Gene set enrichment analysis (GSEA) is a computational method to determine whether a predefined gene set exhibits a consistent and significant difference between two biological states. The genes were ranked based on their degree of differential expression across the two samples, and then the predefined gene set was examined to find whether they were enriched at either the top or bottom of the ranked list. GSEA can detect subtle expression changes. We conducted GSEA analysis using the local version of the GSEA tool available at http://www.broadinstitute.org/gsea/index.jsp (accessed on 15 February 2024). Various datasets, including GO, Reactome, DisGeNET, KEGG, and DO, were used independently for GSEA analysis.

## Figures and Tables

**Table 1:** **Transcription factors up or downregulated by miR-19a.** The table shows an analysis of protein–protein interaction networks of genes up- or downregulated by miR-19a found certain transcription factors, as shown by their rankings.

Rank	TF (miR-19a Upregulated)	TF (miR-19a Downregulated)
1	STAT2	NR4A3
2	MYRF	MEOX1
3	BATF2	CSRNP1
4	NKX62	JUNB
5	BATF3	MEOX2
6	STAT1	EGR2
7	SP100	JUND
8	DBX2	JUN
9	FOXD4L4	EGR3
10	IRF7	TCF21
11	MSC	ATF3
12	ZNF267	RFX8
13	IRF9	TWIST2
14	DUX4	FOS
15	ASCL3	NR4A2
16	FOXS1	LTF
17	MTF1	FOSL1
18	ETV3L	HIF3A
19	GTF2B	NR4A1
20	SP110	TFDP1
21	E2F5	FOXD1
22	ZNF750	GLI1
23	KLF4	BHLHE23
24	GCM2	BNC2
25	HSFY1	HMGA2

**Table 2:** **Transcription factors up or downregulated by miR-19b.** The table shows an analysis of protein–protein interaction networks of genes up- or downregulated by miR-19b found certain transcription factors, as shown by their rankings.

Rank	TF (miR-19b-Upregulated)	TF (miR-19b-Downregulated)
1	NR2E1	CSRNP1
2	OLIG1	JUNB
3	DBX2	MEOX2
4	SOX8	LTF
5	OLIG2	NKX25
6	KCNIP3	MEOX1
7	ARID3C	JUND
8	POU3F3	ZBED6
9	ZBED6	NR4A3
10	NR1H4	HIF3A
11	EMX2	EGR2
12	SOX11	TBX20
13	MLXIPL	ATF3
14	FEZF2	DBX2
15	NR1I2	NKX26
16	FOXG1	JUN
17	IRX6	BHLHE40
18	SCX	NR4A1
19	POU3F2	EGR3
20	CREB3L3	HAND1
21	ZNF804A	HES5
22	LHX2	VSX1
23	PDX1	NR2E1
24	SOX10	RAX
25	RORB	POU3F3

## Data Availability

Data contained within the article.
